# Extracellular vesicles produced in B cells deliver tumor suppressor miR-335 to breast cancer cells disrupting oncogenic programming *in vitro* and *in vivo*

**DOI:** 10.1038/s41598-018-35968-2

**Published:** 2018-12-04

**Authors:** Gonzalo Almanza, Jeffrey J. Rodvold, Brian Tsui, Kristen Jepsen, Hannah Carter, Maurizio Zanetti

**Affiliations:** 1 The Laboratory of Immunology, Department of Medicine and Moores Cancer Center, University of California, San Diego, 9500 Gilman Drive, La Jolla, CA 92093-0815 USA; 2 Division of Medical Genetics, Department of Medicine, University of California, San Diego, 9500 Gilman Drive, La Jolla, CA 92093 USA; 3 IGM Genomics Center, University of California, San Diego, 9500 Gilman Drive, La Jolla, CA 92093 USA

## Abstract

The successful implementation of miRNA (miR) therapies in humans will ultimately rely on the use of vehicles with improved cellular delivery capability. Here we tested a new system that leverages extracellular vesicles (EVs) laden with a tumor suppressor miRNA (miR-335) produced in B cells by plasmid DNA induction (iEVs). We demonstrate that iEVs-335 efficiently and durably restored the endogenous miR-335 pool in human triple negative breast cancer cells, downregulated the expression of the miR-335 target gene *SOX4* transcription factor, and markedly inhibited tumor growth *in vivo*. Remarkably, iEVs-335 mediated transcriptional effects that persisted in tumors after 60 days post orthotopic implantation. Genome-wide RNASeq analysis of cancer cells treated *in vitro* with iEVs-335 showed the regulation of a discrete number of genes only, without broad transcriptome perturbations. This new technology may be ideally suited for therapies aimed to restore tumor suppressor miRNAs in cancer cells, disrupting the oncogenic program established after escape from miRNA control.

## Introduction

Micro-RNAs (miRNAs) are evolutionarily conserved 20–30 nucleotides that represent a large family of gene expression regulators through their ability to prevent translation of specific mRNA into protein^1,2^. Individual miRNAs may repress up to hundreds of transcripts^3^ and can regulate diverse processes including cell growth, metabolism, immunity, inflammation, and cancer. miRNA mutations or mis-expression exist in human cancers suggesting that miRNAs can function either as tumor suppressors or oncogenes (oncomiRs)^4,5^. Consequently, selective miRNA restoration or oncomiR suppression represent new avenues to cancer therapy.

miR-335 is implicated in the growth and metastasis of the triple negative breast cancer cell line MDA-MB-231 derivative 4175 (LM2) cell line^6^. Clinically, triple negative breast cancer patients whose primary tumors have low miR-335 expression have a shorter median time to metastatic relapse^6^. Reportedly, miR-335 inhibits tumor re-initiation but is then silenced by genetic and epigenetic mechanisms^7^. One of the targets of miR-335 is SOX4, a transcription factor involved in embryonic development and cell fate determination^8–10^ and in epithelial to mesenchymal transition (EMT)^11^. *SOX4* expression is elevated in various tumors, including lymphoma, colorectal, cervical, lung, pancreatic, and breast cancer (Human Protein Atlas portal: www.proteinatlas.org). The deregulated expression of this developmental factor has been correlated with increased cancer cell proliferation, cell survival, inhibition of apoptosis, and induction of EMT^12^. Experiments in mice with conditional deletion of *SOX4* in stratified epithelia showed resistance to chemical carcinogenesis leading to delayed onset and tumor size reduction^13^.

Recently, we demonstrated that B cells can be reprogrammed for the enforced biogenesis and synchronous release of short noncoding (snc)RNAs^14^. sncRNAs were packaged and enriched as cargo in extracellular vesicles (EVs) induced in B cells (iEVs), with an estimate content of 3.6 copy number/iEV^15^. Here, we demonstrate that iEVs programmed to contain miR-335 cargo deliver and durably restore miR-335 to LM2 cells, modulate target mRNA expression *in vitro* and *in vivo*, and greatly reduce the growth of orthotopic LM2 tumors in immune deficient NSG mice. Interestingly, regulation was confined to a discrete number of genes, without broad transcriptome perturbations.

## Results

### A plasmid expressing miR-335 doublets in B cells

**Figure 1:**
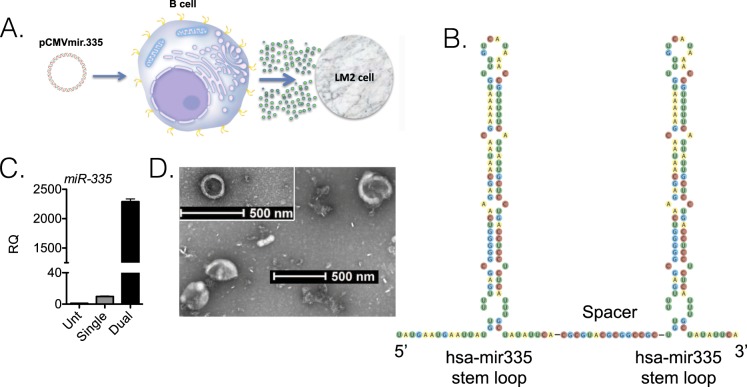
Experimental model and cartoon of dual miR-335 plasmid. (**A**) Schematic of experimental model involving the transfection of murine J558L B cells with pCMVmir.335, the production of induced extracellular vesicles (iEVs), and subsequent treatment on LM2 cells. (**B**) Schematic diagram of tandem hsa-mir335 stem (pre-miR) loops with an intervening spacer sequence. (**C**) Differential abundance of miR-335 in iEVs from J558L cells transfected with pCMVmir.335 containing a single or dual pre-miR-335 sequence, respectively. 10^6^ J558L cells were transfected with 1 µg of plasmid DNA, and the supernatant was collected 48 hrs later. iEVs were isolated by precipitation, counted and analyzed (10^6^) by RT-qPCR amplification using RT-specific primers for miR-335 and SnoRNA202 as a control. RQ (Relative Quantity). Results refer to the mean ± SD of a representative experiment out of three independent experiments. (**D**) Negative staining electron micrographs of iEVs-335. Magnification: Inset (6800x), Main frame 9300x.

At the outset, we reasoned that restoring miR-335 content in LM2 cells would be best achieved by transfecting B cells with a plasmid engineered with two miR-335 precursor stem loops^15^. The general approach and the generation of iEVs in B cells by transfection with plasmid DNA are shown in Fig. 1A. We engineered pCMVmir carrying two pre-miR-335 stem loops in tandem with a nucleotide linker (Fig. 1B). Transfection experiments were performed in the murine myeloma cell line J558L to determine the efficiency of iEVs-335 from cells transfected with a pCMVmir coding for one or two pre-miR-335 stem loops, respectively. miR-335 relative quantification (RQ) in isolated iEVs-335 produced by J558L cells transfected with a single pre-miR-335 stem loop plasmid was modestly increased over control EVs from untransfected J558L cells. In contrast, miR-335 abundance in iEVs-335 produced by J558L cells carrying a pre-miR-335 doublet showed a nearly ~250 fold over that of cells transfected with the singlet (Fig. 1C). Therefore, all subsequent experiments were performed using a pCMVmir carrying two pre-miR-335 stem loops. The iEVs were characterized as having an average size of 100 nm (Supplementary Fig. 1A) and expressing CD63 and CD81 (Supplementary Fig. 1B). Negative staining by electron microscopy shows iEVs to be circular structures of ~ 100 nm diameter with a homogeneous cavity (Fig. 1D). Because of these features iEVs have exosome like characteristics^16^.

### Effects of iEVs containing miR-335 on LM2 cells *in vitro*

**Figure 2:**
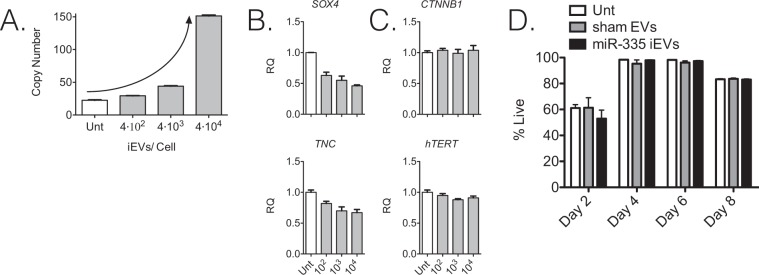
Effects of iEVs-335 on LM2 cells. (**A**) Titration of iEVs-335 input/cell and restoration of endogenous miR-335 content in LM2 cells following co-culture for 48 hrs. Results are expressed as miR-335 copy number/LM2 cell, and refer to the mean ± SD of a representative experiment out of three independent experiments. (**B**) Target modulation of *SOX4* and *TNC* in LM2 cells treated with increasing numbers of iEVs/cell. (**C**) Modulation of bystander genes *CTNNB1* (β-catenin) and human (h)*TERT*. Samples were pre-amplified and then subject to RT-qPCR amplification using RT-specific primers. RQ (Relative Quantity). Results refer to the mean ± SD of a representative experiment out of three independent experiments. (**D**) Viability of LM2 cells treated with iEVs-335 (4 × 10^4^/cell) as compared with untreated (Unt) LM2 cells or LM2 cells treated with sham EVs (4 × 10^4^/cell), by 7-AAD exclusion staining by flow cytometry. Results refer to the mean ± SD of a representative experiment out of three independent experiments. Objects (the syringe, Petri dish and mouse) in panel A are from Openclipart.org (https://openclipart.org/share).

To establish the minimum threshold for effective miR-335 restoration in target LM2 cells, we quantified miR-335 content in LM2 cells incubated *in vitro* for 48 hrs with iEVs-335 over a range of iEVs:LM2 cell ratios (4 × 10^2^–10^4^ iEVs: LM2 cell). The miR-335 copy number increased in a dose dependent manner, with a > 4 fold increase over untreated LM2 cells at the 4 × 10^3^ dose (Fig. 2A). Next, we measured the effect on two miR-335 targets, *SOX4* and tenascin C (*TNC*)^6^. Restoration of miR-335 expression in LM2 cells was associated with a dose dependent reduction in *SOX4* mRNA expression (Fig. 2B). *TNC* expression reduction was less pronounced but also persisted. Two control mRNAs, *CTNNB1* (*β-catenin)* and *hTERT*, which are constitutively expressed in cancer cells, were unaffected, suggesting that the effect on *SOX4* mediated by iEVs-335 was specific (Fig. 2C). Collectively, we concluded that iEVs internalized into LM2 cells release their miR-335 cargo and effectively modulate their target mRNAs, particularly *SOX4*. Treatment with iEVs-335 did not affect LM2 cell viability. LM2 cells were incubated with 4 × 10^4^ iEVs-335:LM2 cells for 48 hours and subsequently cultured under standard culture conditions for an additional 8 days. Cell viability measured by 7-AAD staining did not change in a noticeable way relative to untreated and sham EVs-treated cells (Fig. 2D), suggesting that neither the mere contact/internalization of iEVs nor the cargo content had *per se* an immediate effect on cell survival.

### Suppression of orthotopic tumor *in vivo*

**Figure 3:**
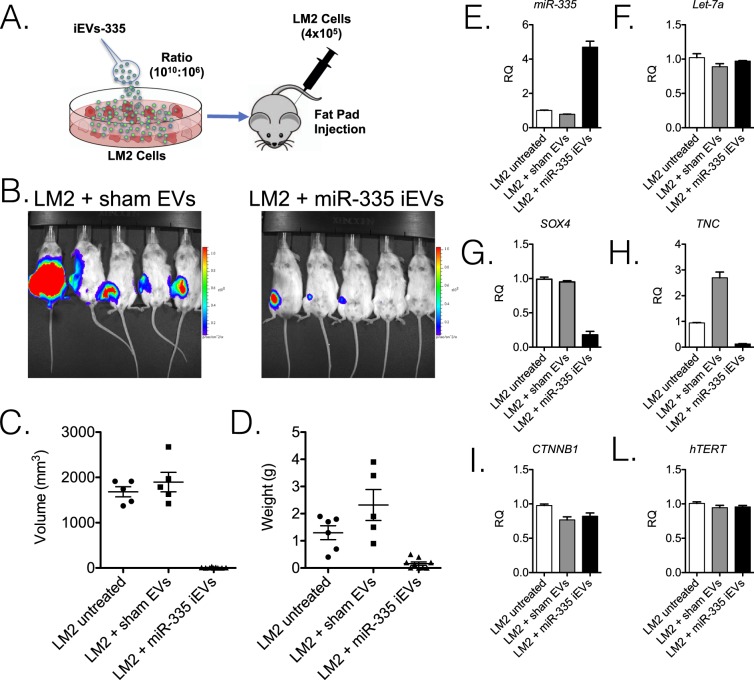
iEVs-335 treatment impedes orthotopic tumor growth in immune compromised mice. (**A**) Schematic representation of the experimental design. LM2 cells were treated by co-culture with iEVs-335 for 48 hrs prior to injection in the mammary fat pad of 10–12 week old NSG mice. Mice were given LM2 cells as one of three groups: untreated (N = 6), pretreated with sham EVs (N = 5), and pretreated with iEVs-335 (N = 9). (**B**) Day 60 bioluminescence images representative of orthotopic tumors formed by LM2 cells treated with either sham EVs (left) or iEVs-335 (right). At sacrifice, volume (mm^3^) (**C**) and weight (g) (**D**) measured for all tumors in the three groups specified in (**A**). (**E**–**L**) RT-qPCR values (RQ) of endogenous miR-335 content (**E**), control miRNA *Let-7a* (**F**), *SOX4* (**G**), *TNC* (H), *CTNNB1* (I), and h*TERT* (L), in explanted tumors born out of untreated LM2 cells, LM2 cells pretreated with sham EVs, and LM2 cells pretreated with iEVs-335 (N = 4). A Grubb’s test was performed to exclude one tumor volume data from the LM2 untreated condition, which was a significant outlier from all other values (Z = 1.9395).

The ability of iEVs-335 to control LM2 tumorigenicity was tested in an orthotopic model by LM2 implantation in the mammary fat pad. Briefly, LM2 cells were incubated with 4 × 10^4^ fold iEVs-335, or control EVs per LM2 cells, for 48 hrs to allow for their uptake/internalization, and the intracellular release of miR-335. NSG mice were then injected in the mammary fat pad with 4 × 10^5^ LM2 cells. Mice were imaged on day 45 and 60, at which point they were sacrificed. Figure 3A outlines the experimental design. On day 45, 4 out of 6 control mice implanted with LM2 cells only, and 5 out of 5 mice implanted with LM2 cells pretreated with sham EVs, had tumors by bioluminescence (not shown). Only 4 out of 9 mice implanted with LM2 cells pretreated with iEVs-335 had tumor. On day 60, all control mice including those implanted with untreated LM2 cells alone and those implanted with LM2 cells pretreated with sham EVs, had large tumors. Upon macroscopic examination all mice had peritoneal invasion and in few instances bone or lymphatic invasion. Among the iEVs-335 group, 4 out of 9 mice had a tumor by *in vivo* imaging but the tumors were considerably smaller than those in mice implanted with LM2 cells treated with sham EVs (Fig. 3B). Local invasion was found in 1 out of 4 tumor-bearing mice only. The average tumor size (mm^3^) was 1,682 ± 250 in the 6 mice given LM2 cells alone and 1,896 ± 479 in sham EVs-treated LM2 cells, respectively. In contrast, the average size (mm) of the four tumors pretreated with iEVs-335 was 7.2 ± 9.8 (Fig. 3C). Likewise, the average weight (g) was 1.3 ± 0.6 in the 6 mice given LM2 cells alone and 2.3 ± 1.2 for tumors from sham EVs treated LM2 cells. The average weight (gr) of tumors treated with iEVs-335 was 0.16 ± 0.18 (Fig. 3D). Thus, pretreatment of LM2 cells with iEVs-335 dramatically impeded tumor growth *in vivo*.

Next, we measured the endogenous levels of miR-335 in the explanted tumors to see if differential tumor growth amongst groups was associated with higher miR-335 levels. The endogenous (RQ) miR-335 value was 1.0 ± 0.06 for the 6 control mice and 0.8 ± 0.02 for tumors pretreated with sham EVs, whereas it was 4.7 ± 0.7 in the four small tumors born out of LM2 cells pretreated with iEVs-335 (Fig. 3E). Endogenous levels of *Let-7a*, a miRNA predicted to be unaffected by iEVs treatment, did not vary between treatment conditions (Fig. 3F). To assess that higher miR-335 levels were functionally relevant, we quantified the mRNA levels of predicted endogenous targets miR-335, *SOX4* and *TNC*, and found them to be considerably reduced compared to those in tumors from all control mice (Fig. 3G–H). Oddly, tumors from sham EVs treated LM2 cells had higher *TNC* expression compared to untreated LM2 cells. No variation was noted in the mRNA levels of two control genes, *CTNNB1* and *hTERT* (Fig. 3I–L). Collectively, the data show effective restoration of the endogenous miR-335 content by iEVs-335 with surprisingly long-lasting effects on target mRNAs regulation in treated cancer cells.

### Durability of miR-335 restoration in LM2 cells

**Figure 4:**
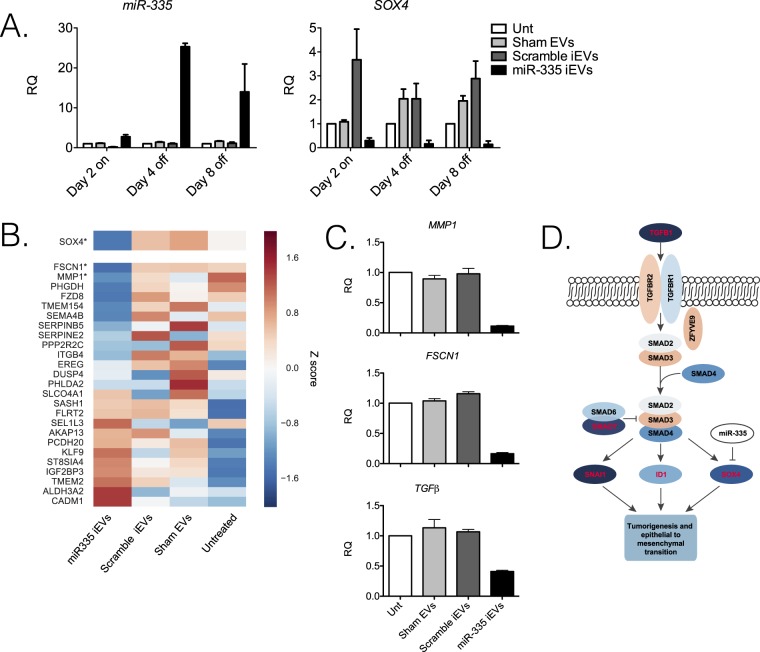
(**A**) Expression of miR-335 and *SOX4* in LM2 cells treated with iEVs-335, scramble iEVs or sham EVs for 2 days (D2 on) and subsequently on day 4 and 8 following removal of iEVs (D4 off and D8 off). (**B**) Whole transcriptome analysis of iEVs-335 treatment of LM2 cells. A heatmap showing relative expression of *SOX4* and target genes in LM2 cells 4 days post-treatment (D4 off) with iEVs-335, iEVs-scrambled, sham EVs and untreated. Log2 gene expression values were converted to z-scores. Color in the heatmap indicates the mean z-score across replicates from the same condition. Genes that are significantly differentially expressed in iEV-335 treated LM2 cells relative to the pooled control conditions are indicated with*. (**C**) Relative quantification (RQ) of *MMP1*, *FSCN1* and *TGFβ* in LM2 cells treated with iEVs-335 4 days after removal of treatment. (**D**) TGFβ signaling via SMAD genes colored according to log 2 fold change in gene expression between miR-335 treated and control conditions. Significantly up- or down-regulated genes in iEVs-335 treated LM2 cells relative to control conditions are indicated by red text.

Tumor growth suppression was associated with a high content of miR-335 and a concomitant reduction of target mRNAs 60 days after iEVs treatment. Because the average miRNA half-life is estimated to be ~5 days^17^, it became important to investigate the longevity of miR-335 restoration/target mRNA regulation by iEVs-335 in LM2 cells. To this end, cultured LM2 cells were treated for 48 hrs as follows: iEVs-335, scrambled miRNA iEVs, and sham EVs, respectively. At the end of a two-day treatment, cells were thoroughly washed and cultured in complete medium for an additional 8 days. Treatment with iEVs-335 resulted in a marked increase in miR-335 content with a peak on day 4. This did not occur in LM2 cells treated with either scrambled miRNA iEVs or sham EVs. Correspondingly, mRNA levels of *SOX4* remained depressed through day 8 (Fig. 4A,B). Significantly, in subsequent *in vitro* experiments, we found higher miR-335 levels through day 50 despite no further manipulations of the cells (Supplementary Fig. 2). To further explore this phenomenon, we performed two experiments. First, we looked at the potential transfer of precursor molecules from pCMVmir(335) transfected iEV-producing J558L cells by probing total RNA with primers specifically designed to anneal a region upstream of the multi-cloning site of pCVMmir(335). The result of this experiment shows no amplification in the iEVs (Supplementary Fig. 3), ruling out the potential carryover of precursor miR-335 molecules from J558L cells to iEVs. Second, we sought to determine whether mature miR-335 in recipient LM2 cells was due the exogenous miR-335 provided by iEVs or was instead contributed by a *de novo* synthesis of miR-335 induced by iEV treatment. To this end, LM2 cells were first treated with aurintricarboxylic acid (ATA), an inhibitor of *de novo* synthesis of miRNA^18^, for 24 or 48 hours. LM2 cells were then washed three times and cultured in fresh cDMEM for 4 or 8 days, with or without the addition of iEVs miR-335. The experimental design is depicted in Supplementary Fig. 4A. ATA inhibited the endogenous production of miR-335 at both 24 and 48 hr time points (Supplementary Fig. 4B,C - left panels). On day 4 and 8 post-ATA treatment, the endogenous production of miR-335 did not increase in ATA-treated LM2 cells that had not been subsequently treated with iEVs miR-335 (Supplementary Fig. 4B,C - right panels). In contrast, LM2 cells treated with iEVs miR-335 expressed mature miR-335 (Supplementary Fig. 4B,C - right panels). This argues strongly against the possibility that mature miR-335 in LM2 cells treated with iEVs may be due to the activation of the biogenesis of endogenous miR-335.

Collectively, these findings suggest that the uptake of iEVs with a miR-335 payload leads to restoration of the endogenous miR-335 pool beyond the average half-life of miRNA. Since our findings do not substantiate *de novo* biogenesis of endogenous miR-335, the observed long-lasting effects may then be due to a combination of slow intracellular release and protection from degradation.

### RNASeq analysis of LM2 cells treated with iEVs-335

RNASeq interrogation was performed to assess effects on the transcriptome in LM2 cells after treatment with iEVs-335. Transcriptome profiles were compared from three replicates each of iEVs-335, iEVs scrambled, sham EVs and untreated LM2 cells. We performed unsupervised clustering of whole-transcriptome profiles and observed very high correlation across treatment conditions, with the highest correlation observed among samples treated with iEVs-335, suggesting small but consistent differences in gene expression relative to the control conditions (Supplementary Fig. 5). As predicted, *SOX4* expression was down-regulated in LM2 cells treated with iEVs-335 relative to pooled control conditions (untreated, iEVs-scrambled and sham EVs) (Fig. 4B; and Supplementary Fig. 6). However, *SOX4* target genes as a whole were not significantly differentially expressed across conditions although two of the genes, *MMP1* (Matrix metalloproteinase-1) and *FSCN1* (Fascin Actin-Binding Protein 1), were significantly down-regulated in response to treatment with iEVs-335 (Fig. 4B, Supplementary Table 1). The down-regulation was confirmed by RT-qPCR (Fig. 4C). Aside from *SOX4*, only two other miR-335 target genes were significantly down regulated, *FOXA2* and *DKK1* (Supplementary Fig. 6, Supplementary Table 2).

Exploratory analysis of differentially expressed (DE) genes revealed overall 14% of expressed protein coding genes (1739 out of 12134) were affected by iEVs-335 treatment at a 5% false discovery rate, and only 5.7% (696) had a fold change of at least 1.5. DE genes were predominantly down-regulated by iEVs-335 treatment (Supplementary Fig. 7; Supplementary Table 3) and enrichment analysis implicated a small number of pathways associated with cellular signaling (TGFβ, TNFα and mTOR), energy metabolism (cholesterol homeostasis and hypoxia), and differentiation (myogenesis) (Supplementary Table 4). We also performed weighted gene co-expression network analysis^19^ to identify networks of genes with highly correlated expression. Although WGCNA analysis detected two clusters of co-expressed genes, these clusters were only weakly correlated with miR-335 treatment (rho ~0.15; p < 0.65).

The TGFβ pathway has previously been implicated in breast cancer stem cell maintenance and EMT transition^20^ via transcriptional regulation of genes including transcription factor *SOX4*, and transcriptional repressors *ID1*^21^ and *SNAI1*^22^. All three of these genes were significantly down-regulated in iEVs-335 treated LM2 cells (Fig. 4D), as was *TGFβ1* itself. Thus, treatment with mir-335 may also indirectly down-regulate production of *SOX4 via* a reduction in TGFβ signaling. The combined down-regulation of *TGFβ1* and downstream targets *SOX4*, *ID1* and *SNAI1* suggests that the reduced ability of miR-335 treated LM2 cells to generate tumors in mice may be in part due to loss of TGFβ signaling.

## Discussion

Here we show that iEVs generated in B cells and enriched in miR-335 can be used to restore miR-335 endogenous content in triple negative cancer cells that have a reduced content, enabling the regulation of SOX4, a transcription factor that is overexpressed in cancer cells. The internalization of iEVs-335 in LM2 cells was effective at down-regulating *SOX4* mRNA *in vitro* and *in vivo*. We verified that these effects were not restricted to LM2 cells as other breast cancer cell lines, ovarian and pancreatic cell lines, were also similarly affected (Supplementary Fig. 8). Surprisingly, tumors born out of LM2 cells treated *ex vivo* with iEVs-335 showed a profound *SOX4* downregulation sixty days after orthotopic implantation, suggesting that *SOX4* negative regulation by a single treatment iEVs-335 is long-lasting.

The mechanism for the durable effects of iEVs treatment is presently unknown. The fact that the viability of LM2 cells treated with iEVS-335 was consistently preserved up to day 8 in culture argues against a direct apoptotic or toxic effect. Rather, it suggests that reconstitution of miR-335 in target cells triggered durable gene regulation, such as that of genes involved in tumor growth and local invasion. Figure 4A showed a selective downregulation of *SOX4* during the first week after treatment. RNASeq analysis on day 4 post-treatment (Fig. 4B) revealed a selective perturbation of the *SOX4* transcriptional network with a significant downregulation of two genes *MMP1* and *FSCN1* (Supplementary Table 1) even though other genes were also affected without reaching, however, the level of significance. MMP1, a collagenase that cleaves collagen Type I, II, III, VII and X, is overexpressed in a variety of cancers types including breast cancer and in circulating cancer cells^23^. Fascin1, a member of the cytoskeleton protein family, has been reported to regulate invasion of breast and pancreatic cancer cells by increasing cell motility^24,25^. Interestingly, neither the RNASeq analysis not a RT-qPCR could reveal a statistically relevant perturbation of *TMEM2*, a transmembrane protein gene reported to be a SOX4 target (Supplementary Fig. 9)^26^. We also observed down-regulation of other hallmark cancer genes^27^ in the miR-335 treated condition relative to control (Supplementary Table 5), in particular genes associated with cell cycle, apoptosis and phosphorylation (kinase signaling) hallmarks (p < 0.05). Furthermore, we generated a Volcano plot that illustrates that there is a bias for hallmark associated genes (in red) to have significant decreased expression in association with miR-335 treatment (Supplementary Fig. 10). Consistent with the idea of the activation of a regulatory cascade is a report showing that miR-335 may exert its tumor-controlling effects via upregulation of BRCA1 mRNA^28^.

RNA sequencing analysis suggested that overall, treatment with iEVS-335 had a modest effect on gene expression. While expression profiles across samples were highly correlated (Supplementary Fig. 5), samples treated with iEVs-335 showed even higher correlation, consistent with a small number of differences in gene expression between treated and control samples. Only three miR-335 target genes were significantly down-regulated (*SOX4*, *FOXA2* and *DKK1*) and downstream of *SOX4*, only *FSCN1* and *MMP1* showed a significant decrease in expression. When pathway level enrichment for differentially expressed genes was assessed, only a small number of biological pathways were detected. These pathways included TGFβ, TNFα and mTOR signaling, pathways that regulate cell growth, proliferation, differentiation, apoptosis and epithelial to mesenchymal transition. Thus, a small perturbation in gene expression by miR-335 treatment was associated with specific down-regulation of key pathways required for tumorigenesis.

Remarkably, a one-time internalization of iEVs-335 proved to be efficient to suppress and in some instances block, orthotopic tumor formation, confirming the tumor-suppressor function of miR-335. A possible interpretation is that once internalized into LM2 cells, iEVs-335 degrade slowly, releasing their payload over time, leading to selected gene perturbations that, as demonstrated herein, relate to genes of the primary mRNA target transcriptional network, as well as other genes with participatory role such as TGFβ, which drives malignant progression, invasiveness and dissemination^29^. In addition to their slow release and intracellular degradation, iEVs proved to be superior to equimolar concentrations of soluble miR-335 mimics in their ability to downregulate the *SOX4* mRNA target (Supplementary Fig. 11). Expression of miR-335 is reduced in various cancer types in humans besides triple negative breast cancer^30–35^, and low miR-335 expression levels have been associated with reduced recurrence-free survival, representing an independent indicator of poor overall survival^7,32^. As demonstrated here, iEVs-335 could lend themselves as a new form of therapeutic intervention in cancers in which genomic interrogation documents a decrease of tumor suppressor miR-335 and/or an increase of *SOX4*. Optimization of iEVs as vehicles of miRNA therapies in general will further require the addition of simple, cost-effective modalities for precision tissue targeting *in vivo* upon systemic administration to enhance therapeutic efficacy while reducing off-target effects.

## Material and Methods

### Mice

8–10 week old NOD *scid* gamma (NSG) mice were purchased from The Jackson Laboratories.

### Cell lines and chemicals

MDA MB 231–4175 (LM2) cells are human TNBC cells derivative of MDA-MB 231 cells stably transduced with a lentivirus expressing a triple-fusion reporter (abbreviated “TGL”) encoding herpes simplex virus thymidine kinase 1, green florescence protein and firefly luciferase^36^. LM2 cells were kindly obtained from the Cell Repository of the Memorial Sloan-Kettering Cancer Center (New York, NY). MDA-MB 231, SKBr3, SKOV3, and PANC1 cells were purchased from the American Type Cell Collection. Aurintricarboxylic acid (ATA) was purchased from Sigma-Aldrich.

### Plasmid Constructs

A dual miRNA construct containing miR-335-miR-335 was synthesized with unique SgfI/XhoI ends by Integrated DNA Technologies (IDT, Coralville, IA). A single miRNA (miR-335) scramble (uuguauuauuuuuaacauaugaaugaauua) construct was similarly synthesized with unique SgfI/XhoI ends. Constructs were cloned into the pCMVmir (Origene, Rockville, MD) expression vector by digesting with SgfI and XhoI, and subsequent ligation of the insert into the pCMVmir vector. The ligation mixture was transformed into TOP10 competent cells (Life Technologies, Carlsbad CA). Transformed cells were plated, and clones were selected and grown overnight at 37 °C. DNA was extracted with Promega Wizard Plus SV Minipreps DNA Purification System (Promega, Madison WI). The resulting plasmid was termed pCMV dual mir335. The insert was verified via sequencing. The plasmid was stored at −20 °C until transfection. Single miRNA construct containing miR-335 was generated through excision from the dual miRNA construct by digestion and ligation using unique restriction sites (SgfI-MluI or NotI-XhoI) within the minigene to yield pCMV miR-335. The correctness of each plasmid construct was verified by sequencing.

### Cell Culture and Transfection

J558L mouse B cell myeloma cells were grown in suspension in cRPMI with 10% fetal bovine serum (FBS). Cells were grown to 80% confluence. 2 × 10^6^ cells were transfected with 1 μg of pCMVmiR plasmid utilizing the Lonza VACA-1003 transfection kit V in a Nuclefector 2b device (Lonza, Walkersville, MD). Cells were allowed to recover in a T25 flask upright at 37 °C with 5% CO_2_ for 48 hrs. In experiments in which sncRNA copy number was determined, transfected J558L cells were cultured in EXO-FBS-50A-1 exosome-depleted FBS (Exo-FBS, Systems Biosciences, Mountain View, CA).

### EV Isolation and enumeration

Forty-eight hours post-transfection 1 mL of culture supernatant was collected and incubated with 0.5 mL of Total Exosome Isolation solution (Life Technologies, Carlsbad, CA) for 1 hr at room temperature. The EV-containing mixture was spun at 16,000 RPM at 4 °C for 1 hr. The EV pellet was resuspended in 100 µL of PBS at room temperature and stored in 1.5 mL Eppendorf tubes at −20 °C until use. EVs isolated from untransfected or sham transfected (electroporated only) J558L cells served as a control.

The number of vesicles recovered was determined by Nanoparticle Tracking Analysis (NTA) on a NanoSight LM-10HS equipped with a 405 nm laser (NanoSight, Wiltshire, UK) calibrated with polystyrene latex microbeads at 100 nm and 200 nm prior to analysis. Resuspended vesicles were diluted 1:100-1:300 with PBS to yield 20–100 objects per frame. iEVs were manually injected into the sample chamber at room temperature. Each sample was measured in triplicate at camera setting 14 with acquisition time of 30 seconds and detection threshold setting of 7. At least 200 completed tracks were analyzed per video. The NTA analytical software version 2.3 was used for capturing and analyzing the data.

### Negative staining electron microscopy

A morphological analysis of iEVs was performed by standard negative stain method. Briefly, Formvar-carbon-coated copper grids (100 mesh, Electron Microscopy Sciences, Hatfield, PA) were placed on 20 μl drops of each sample solution displayed on a Parafilm sheet. After allowing material to adhere to the grids for 10 minutes, grids were washed x3 by rising through 200 μl drops of milli-Q water before being left for 1 min on 2% (wt/vol) uranyl acetate (Ladd Research Industries, Williston, VT). Excess solution was removed with Whatman 3MM blotting paper, and grids were left to dry for a few minutes before viewing. Grids were examined using a Tecnai Spirit G2 BioTwin transmission electron microscope operating at 80 kV. Images were recorded using an Eagle 4 K digital camera.

### Western blot analysis

1 × 10^6^ EVs for each condition shown in Supplementary Fig. 1B were processed for Western blot analysis as follows. iEVs were counted by NTA and lysed at 70 °C for 5 minutes in 5 × loading dye buffer in a total volume of 20 µL. 15 µL samples were loaded into a 4–20% mini protean TGX gel (Bio-Rad). Precision Plus Protein Standard was used as a marker for weight (Bio-Rad). Gel was ran at 100 volts for 75 minutes and then transferred to 0.2 µm PVDF membrane using a Trans Blot Turbo device (Bio-Rad). Wester blotting protocol from Cell Signal Inc. was followed for antibody detection. Primary antibodies rat anti-mouse CD63 monoclonal antibody (Cat#564222, BD Biosciences) and hamster anti-mouse CD81 monoclonal antibody (Cat#559519) were both used at 1:1,000 dilution. Horseradish peroxidase-labeled rabbit antibodies to rat/hamster IgG were used at 1:2,500 dilution. Bound antibodies were revealed with Clarity Western ECL substrate (Bio-Rad). Blot was exposed for 10 minutes with Blue Devil autoradiography film (Genesee Scientific).

### RNA Extraction and Copy Number Determination

1 × 10^6^ transfected or untransfected J558L cells, and 1 mL of culture supernatant, were collected for RNA extraction using ZYGEM RNAtissue Plus System (Zygem, Hamilton, NZ) according to the manufacturer’s protocol. RNA from cell supernatant (200 µL) was extracted with the Qiagen miRNeasy Serum/Plasma kit following the manufacturer’s protocol. EVs extraction was performed using the ZYGEM RNAtissue Plus System.

cDNA was generated from intracellular and iEV miRNA with Taqman small RNA assays. Input RNA was normalized to 100 ng/sample for intracellular and exosome RNA, and to 25 ng/sample for extracellular miRNA. Taqman MicroRNA Reverse Transcription Kit was utilized for all samples per manufacturer’s instructions. Cycling conditions for qPCR were: 40 cycles, 96 °C denature 30 secs, 60 °C anneal/extension 30 secs. Results are expressed as RQ (Relative quantity of sample) that was calculated using the formula: Relative Quantity_target_ = E_target_ (Cq (control) − Cq (treatment)). Abbreviations: E = Efficiency of primer set; C_q_ (control) = Average C_q_ for the control or untreated sample; C_q_ (treatment) = Average C_q_ for treated sample; Target = The gene of interest or reference gene.

Copy number was determined in samples normalized at 100 ng cDNA/reaction run concomitantly with a standard curve constructed with known amounts (100–0.01 ng) of miR-335 cDNA and an endogenous control standard curve constructed using known amounts (100–0.01 ng) of snoRNA202 cDNA (Applied Biosystems snoRNA202 – assay No. 001232 - specific reverse transcription primers). Samples were run in duplicate. Relative expression was determined by the Ct value of test samples vs. the endogenous control. Once the amount (ng) of specific target was determined, the copy number present in each reaction was calculated using the following formula: (ng × 6.0223 × 10^23^)/(number of nucleotides × 1.0 × 10^9^ × 650) as indicated in http://www.uic.edu/depts/rrc/cgf/realtime/stdcurve.html. Copy number/EV determination was calculated as follows: [Total copy number/No. EVs sample].

### Treatment of LM2 cells with iEVs and *in vivo* studies

LM2 cells were plated at 1 × 10^6^ and treated with iEVs at 4 × 10^4^ iEVs:LM2 cell ratio for 48 hrs. After treatment cells were washed x3, and resuspended in PBS until implanted (4 × 10^5^) into the right mammary fat pad in 50 μl. Mice were monitored for tumor take by palpation. When tumors became palpable, tumor size was determined through two-dimensional caliper measurements every three days. On day 30 and prior to sacrifice on day 60 mice received 6 mg of D-luciferin in PBS i.p., allowed to rest for 6 minutes, and imaged in a Xenogen IVIS system. At sacrifice tumors were resected, weighed and measured by caliper. Tumor volume was calculated using the ellipsoid formula: V = ½ (H × W^2^). All animal work was approved by the UCSD Institutional Animal Use and Care Committee.

### RNA sequencing

Triplicate RNA samples from LM2 cells corresponding to four different conditions as shown in Fig. 4A,B were sequenced to a targeted 25 million reads per sample. Sailfish v0.7.4 was used to estimate transcript abundance as transcripts per million (TPM) from single end 75 bp sequencing reads. Transcript levels were log2 transformed, and any genes with mean TPM <1.0 were excluded from further analysis.

Samples were clustered by transcriptome profile using agglomerative hierarchical clustering with Euclidean distance and single linkage, and Pearson correlation was calculated between pairs of samples. Differential expression was determined by comparison of the iEVs-335 treated condition to the pooled control conditions. Individual genes were assessed for differential expression using a *t*-test and multiple testing correction was performed using the Benjamini-Hochberg method.

Twenty-six SOX4 target genes identified by anti-SOX4 anti-body were obtained from Lee *et al*.^26^. Overall enrichment of the 26 gene set for differentially expressed genes was evaluated using Fisher’s exact test. Mir-335 target genes were obtained from mirTarBase^37^, and only targets supported by strong experimental evidence were retained (Supplementary Table 2).

We preformed exploratory gene set enrichment analysis (GSEA)^38^ using the stand-alone software (v3.0) to identify other biological activities perturbed by miR-335. GSEA was run with default parameters on three MSigDB collections: c2 (curated gene sets), c6 (oncogenic signature) and h (hallmark gene sets). A false discovery rate of 0.05 was used as the cutoff to consider a gene set as enriched for differentially expressed genes. No gene sets in the c2 or c6 groups met the significance criteria. WGCNA was run on whole transcriptome profiles with default parameters using the WGCNA R package v1.61^19^.

### Precursor miR-335 analysis

1 × 10^5^ LM2 cells (treated with iEV mir335) and J558L transfected with pCMVmir335 were extracted with Qiagen DNA blood mini kit. 1 × 10^6^ miR-335-laden iEVs were also processed with a Qiagen DNA blood mini kit. 50 ng of nucleic acid or volumetric equivalent were used in each 20 µL reaction and allow to anneal with primers pCMV ins chk F 5′-TTGTAATACGACTCACTATAGG−3′ and pCMV ins chk R 5′-GGATCTGTTCAGGAAACAGC-3′. Thermocycling parameters were as follows: 96 °C 10 min, 30 cycles of 96 °C denaturing, 53.7 °C annealing and 72 °C extension.

## Electronic supplementary material

SUpplementary Info

Supplementary Data Set

Supplementary Data Set
