# Supplementary material for: Extracellular vesicles produced in B cells deliver tumor suppressor miR-335 to breast cancer cells disrupting oncogenic programming in vitro and in vivo
Source: Sci Rep. 2018 Dec 4;8:17581. doi: 10.1038/s41598-018-35968-2 (PMC6279829; doi:10.1038/s41598-018-35968-2)
Supplement: Supplementary file 1 — SUpplementary Info [file 41598_2018_35968_MOESM1_ESM.pdf]

SUPPLEMENTARY DATA

Extracellular vesicles produced in B cells deliver tumor suppressor miR-335 to breast cancer cells disrupting oncogenic programming *in vitro* and *in vivo*

Almanza, Rodvold, Tsui, Jepsen, Carter and Zanetti

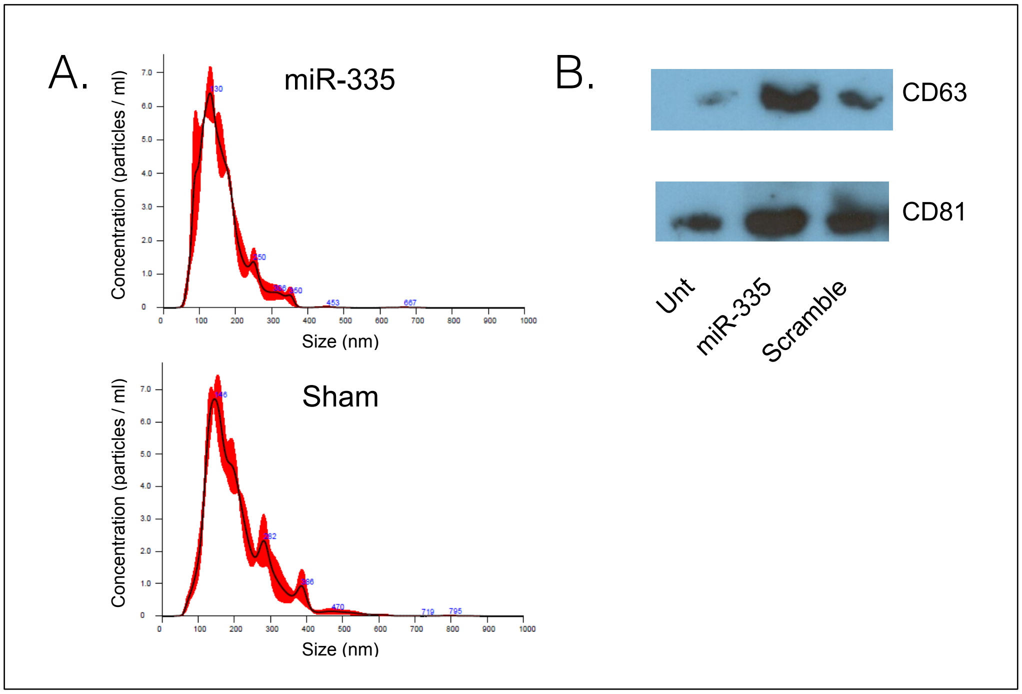

Supplementary Figure 1. Characterization of iEVs. (**A**) iEV's produced from J558L cells were counted in a Nanosight NTA device as described in Material and Methods. 1x10^{6} cells were transfected with pCMV miR-335. Transfected J558L cells cultured in exosome-depleted cDMEM. Supernatants were collected 48 hrs post-transfection. iEVs precipitated with Total Exosome Isolation Solution as described in Material and Methods. All counting was done according to manufacturer's guidelines. (**B**) Western Blot analysis of iEVs for expression of CD63 and CD81. iEVs from non-transfected cells (Unt), miR-335 transfected, and scramble miR transfected J558L cells, were run on 4-12% PAGE gradient gel under non-denaturing conditions. After transfer to 0.2 µm PVDF membrane, the blotted bands were incubated with a rat anti-mouse CD63 and a hamster anti-mouse CD81 monoclonal antibody, that were revealed with a horseradish peroxidase-labeled rabbit antibodies to rat/hamster IgG. Lane 1: untreated (Unt) iEVs 2: iEVs miR-335, lane 3: iEVs scramble miR.

SUPPLEMENTARY DATA

Extracellular vesicles produced in B cells deliver tumor suppressor miR-335 to breast cancer cells disrupting oncogenic programming *in vitro* and *in vivo*

Almanza, Rodvold, Tsui, Jepsen, Carter and Zanetti

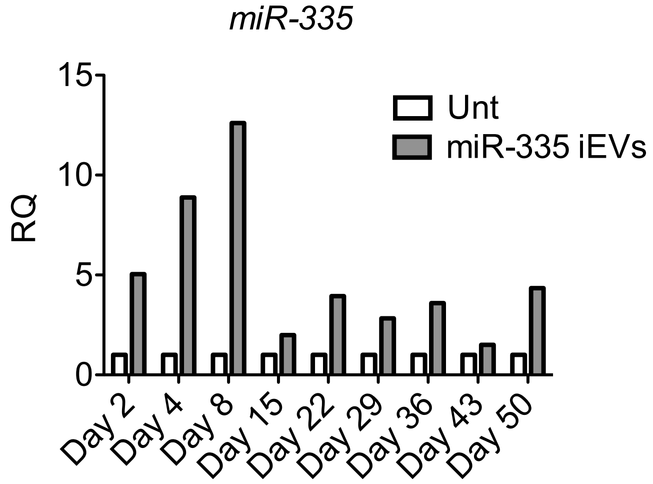

Supplementary Figure 2. iEVs-miR335 durably affect *miR-335* expression. LM2 cells were untreated (Unt) or treated with iEVs produced by B cells transfected with miR-335 constructs (4e10^4 iEVs/LM2 cell) for two days and harvested at respective time points up to day 50. Each condition was then analyzed by RT-qPCR simultaneously to determine expression of *miR-335* after normalizing to the untreated condition for that day. Gene expression was normalized to *snoRNA202.*

SUPPLEMENTARY DATA

Extracellular vesicles produced in B cells deliver tumor suppressor miR-335 to breast cancer cells disrupting oncogenic programming *in vitro* and *in vivo*

Almanza, Rodvold, Tsui, Jepsen, Carter and Zanetti

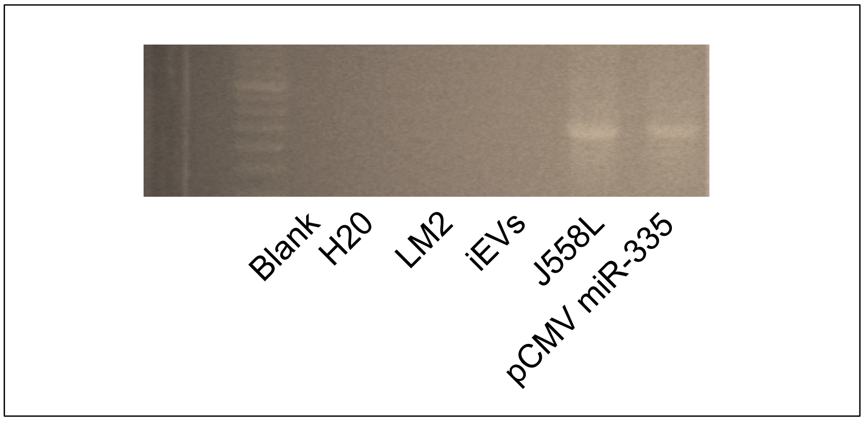

Supplementary Figure 3. PCR detection of precursor miR-335. For method details see Material and Methods. Lane 1: 100bp ladder; lane 2: blank; lane 3: H2O; lane 4: LM2; lane 5: miR-335 iEV's; lane 6: transfected J558L cells; lane 7: pCMV miR-335 (positive control).

SUPPLEMENTARY DATA

Extracellular vesicles produced in B cells deliver tumor suppressor miR-335 to breast cancer cells disrupting oncogenic programming *in vitro* and *in vivo*

Almanza, Rodvold, Tsui, Jepsen, Carter and Zanetti

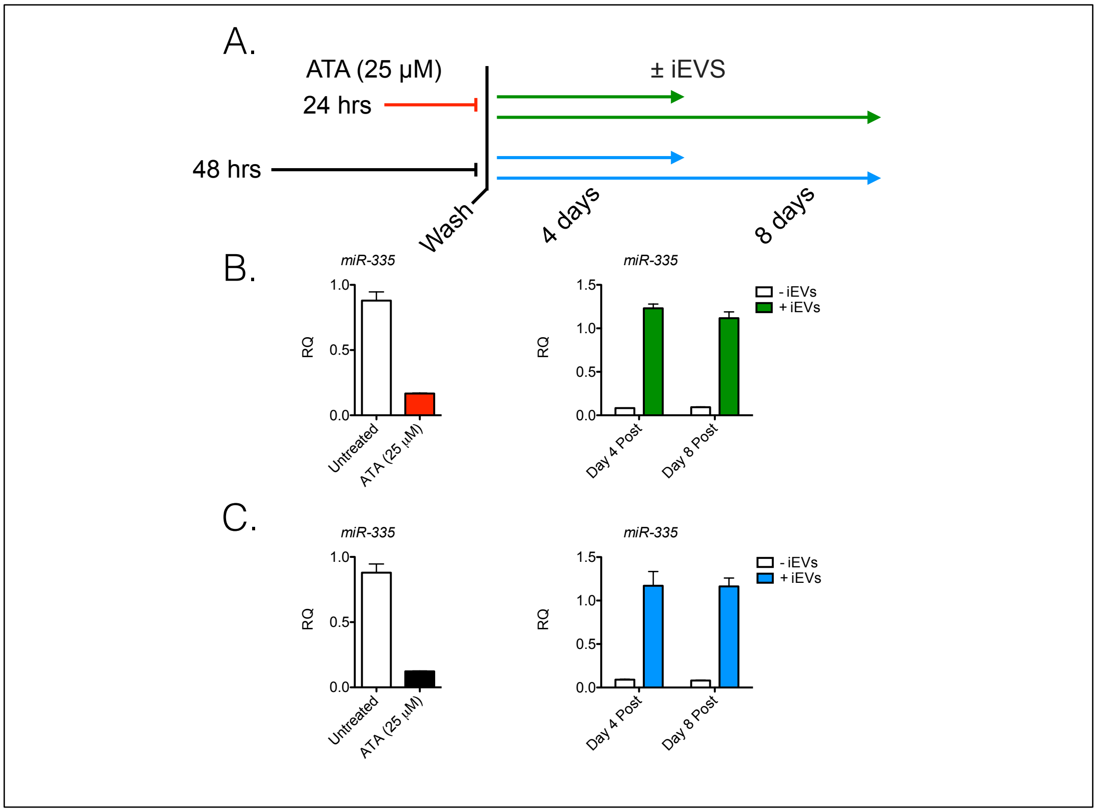

Supplementary Figure 4. Persistence of exogenous miR-335 in LM2 cells. LM2 cells were first treated with aurintricarboxylic acid (ATA), an inhibitor of de novo synthesis of miRNA [Tan, 2012 #7611], for 24 or 48 hours. LM2 cells were then washed three times and cultured in fresh cDMEM for 4 and 8 days with or without addition of iEVs miR-335. The experimental design is depicted in panel A. ATA inhibits the endogenous production of miR-335 at both 24 and 48 hours (B and C - left panels). On day 4 and 8 the endogenous production of miR-335 did not increase in ATA treated LM2 cells that had not been treated with iEVs (B and C - right panels). In contrast, LM2 cells treated with iEVs miR-335 expressed mature miR-335 (B and C - right panels). Technical details are provided in Material and Methods.

SUPPLEMENTARY DATA

Extracellular vesicles produced in B cells deliver tumor suppressor miR-335 to breast cancer cells disrupting oncogenic programming *in vitro* and *in vivo*

Almanza, Rodvold, Tsui, Jepsen, Carter and Zanetti

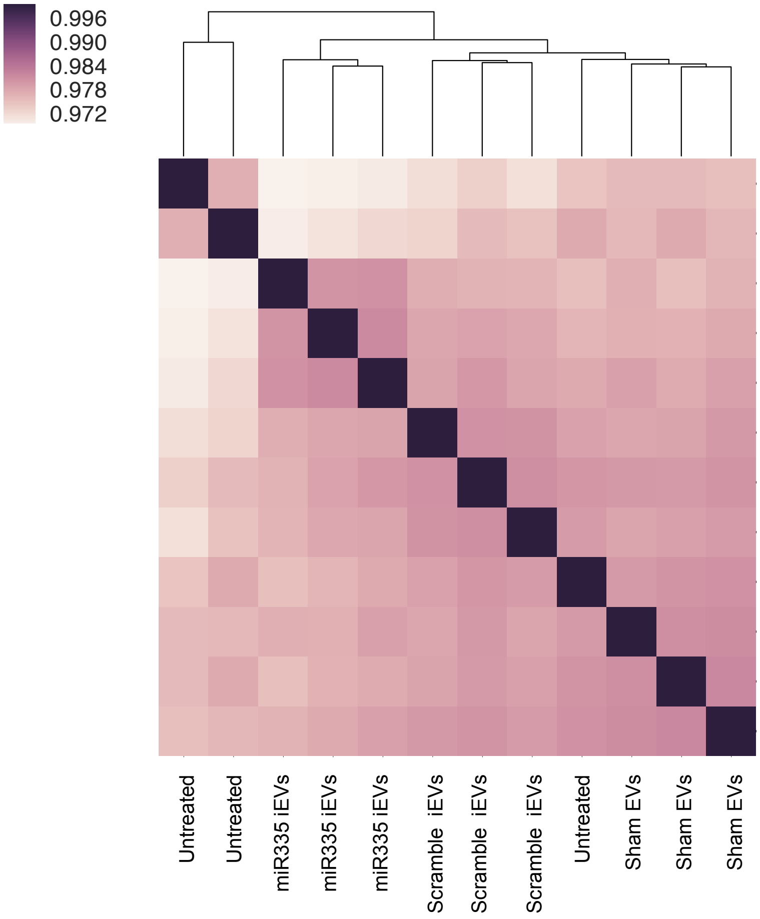

Supplementary Figure 5. Transcriptome-based clustering of iEVs-335 and control LM2 conditions. Replicate samples of LM2 cells tended to cluster according to treatment status. Overall correlation amongst samples was high. Transcriptome profiles of iEV-335 treated LM2 replicates were more correlated with one another than with control samples suggesting the presence of small but consistent transcriptional differences.

SUPPLEMENTARY DATA

Extracellular vesicles produced in B cells deliver tumor suppressor miR-335 to breast cancer cells disrupting oncogenic programming *in vitro* and *in vivo*

Almanza, Rodvold, Tsui, Jepsen, Carter and Zanetti

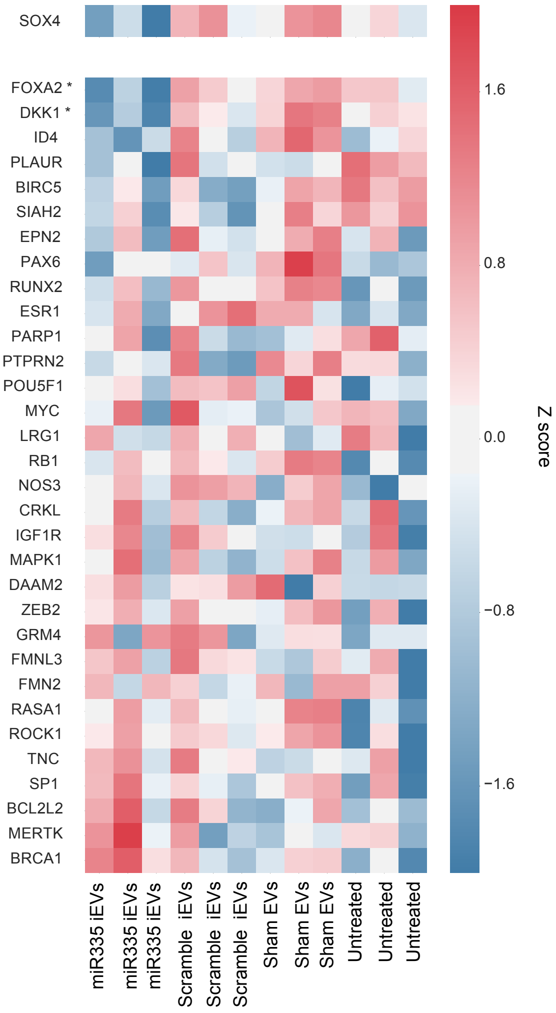

Supplementary Figure 6. Impact of iEV-335 on expression of miR-335 target genes. Heatmap showing z-scored log2 expression of miR-335 target genes across iEVs-335 and control conditions. Genes significantly differentially expressed in iEVs-335 treated LM2 cells relative to pooled control conditions are indicated with *.

SUPPLEMENTARY DATA

Extracellular vesicles produced in B cells deliver tumor suppressor miR-335 to breast cancer cells disrupting oncogenic programming *in vitro* and *in vivo*

Almanza, Rodvold, Tsui, Jepsen, Carter and Zanetti

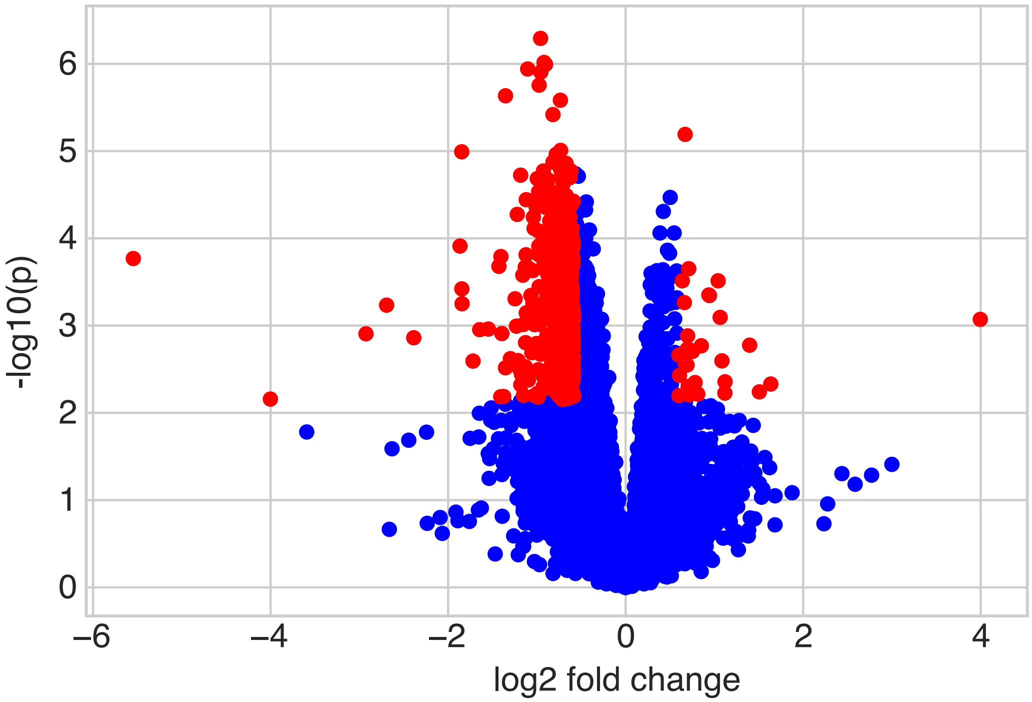

Supplementary Figure 7. Summary of differentially expressed genes in LM2 cells treated with iEV-335 relative to pooled control conditions. Volcano plot showing log2 (fold change) relative to –log 10 (p-value). Colored points indicate genes with at least a 1.5 fold change in expression that were also significant after multiple testing correction. Overall, the significantly differentially expressed genes were biased toward down-regulation.

SUPPLEMENTARY DATA

Extracellular vesicles produced in B cells deliver tumor suppressor miR-335 to breast cancer cells disrupting oncogenic programming *in vitro* and *in vivo*

Almanza, Rodvold, Tsui, Jepsen, Carter and Zanetti

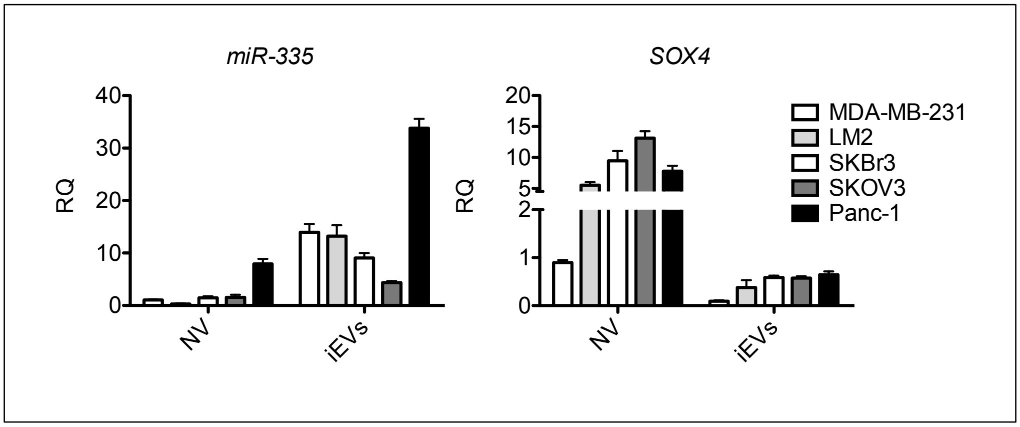

Supplementary Figure 8. Endogenous levels of miR-335 and Sox4 in various cancer cell lines with or without treatment with iEVs miR-335. Tumor cell lines were treated with iEVs miR-335 for 48 hrs at $4x10^4$ iEVs:LM2 cell ratio. Total RNA was harvested and processed as described in Material and Methods. RQ (Relative Quantity) refers to RT-qPCR amplification using RT-specific primers for miR-335 and SnoRNA202 as a control (upper panel) and *SOX4* transcription (lower panel). Results refer to the mean ± SD of triplicate wells.

Extracellular vesicles produced in B cells deliver tumor suppressor miR-335 to breast cancer cells disrupting oncogenic programming *in vitro* and *in vivo*

Almanza, Rodvold, Tsui, Jepsen, Carter and Zanetti

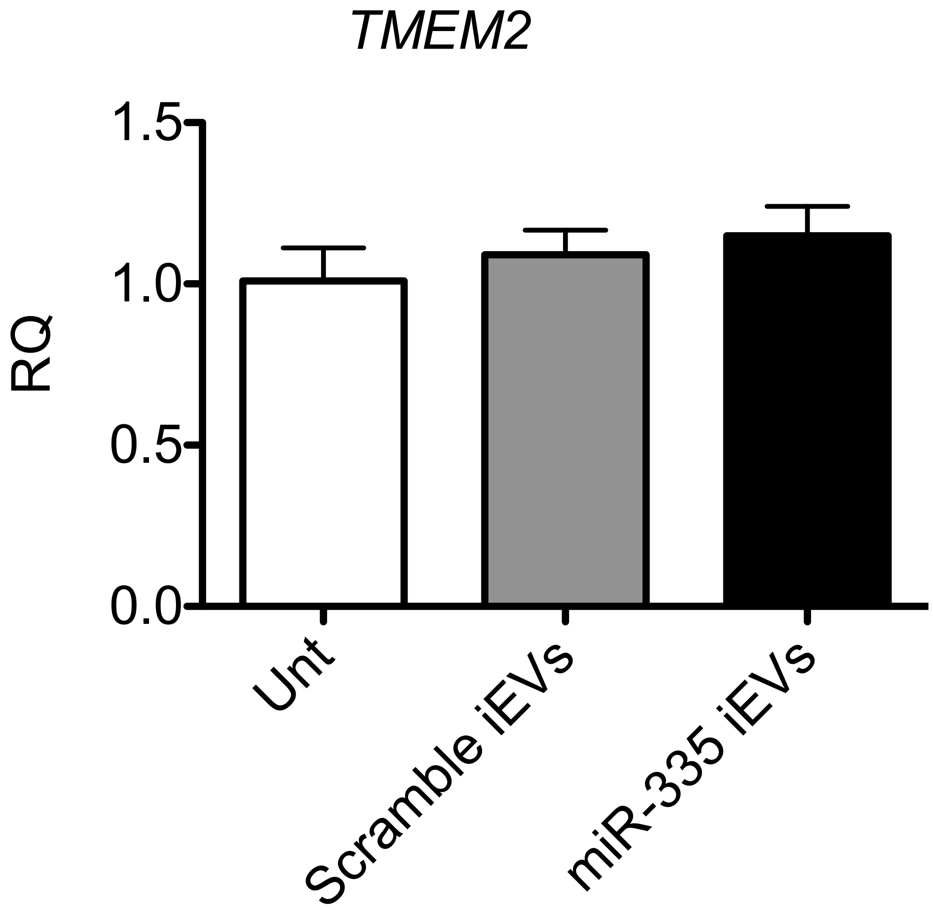

Supplementary Figure 9. iEVs-miR335 does not affect *TMEM2* expression. LM2 cells were untreated (Unt) or treated with iEVs produced by B cells transfected with control scramble or miR-335 constructs ($4e10^4$ iEVs/LM2 cell) for two days and allowed to rest for a subsequent four days. Each condition (n=3) was then analyzed by RT-qPCR to determine expression of *TMEM2.* Relative quantification (RQ) was determined by normalizing expression to untreated condition. Endogenous control was *β-actin.* Error bars are representative of SEM.

SUPPLEMENTARY DATA

Extracellular vesicles produced in B cells deliver tumor suppressor miR-335 to breast cancer cells disrupting oncogenic programming *in vitro* and *in vivo*

Almanza, Rodvold, Tsui, Jepsen, Carter and Zanetti

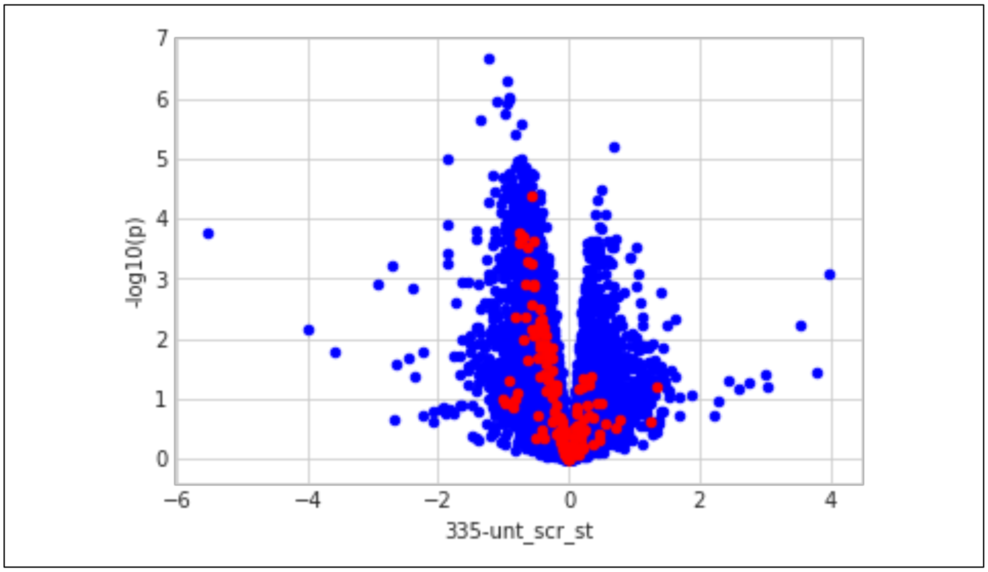

Supplementary Figure 10. Volcano plot showing the differential expression of hallmark associated genes (red) between miR-335 treated and control conditions.

SUPPLEMENTARY DATA

Extracellular vesicles produced in B cells deliver tumor suppressor miR-335 to breast cancer cells disrupting oncogenic programming *in vitro* and *in vivo*

Almanza, Rodvold, Tsui, Jepsen, Carter and Zanetti

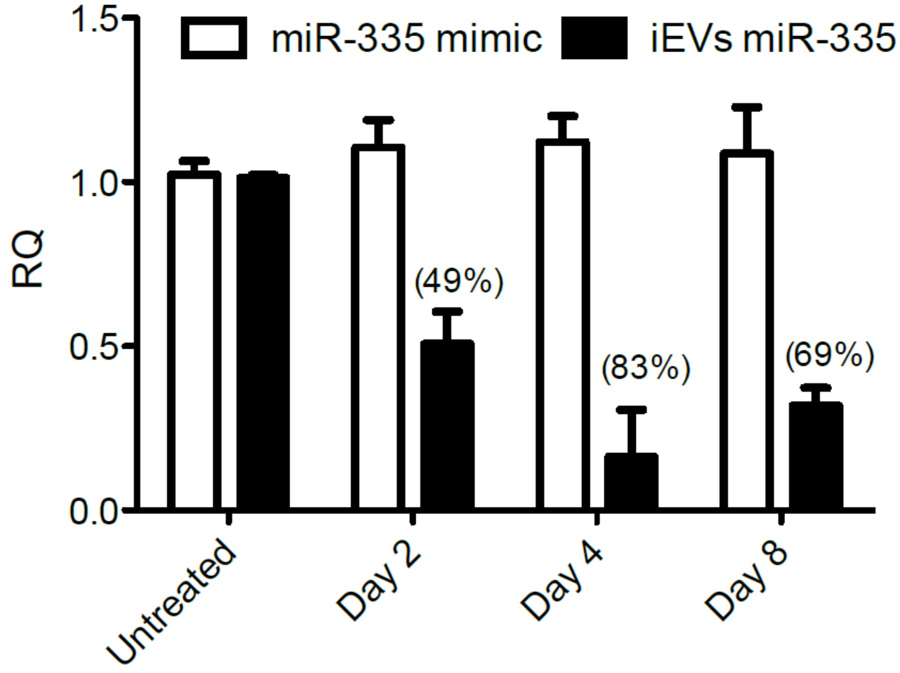

Supplementary Figure 11. **Comparative analysis of SOX4 mRNA levels following treatment with HSA-mir-335 mimic or iEVs-335**. 1x10^5 LM2 cells were plated in T25 flasks. LM2 cells were treated with 100 ng of HSA-mir-335 mimic (Millipore-Sigma, HMI0490-5NMOL). 3x10^4 copies/cell of soluble HSA-mir-335 mimic were added based on equivalent number of copies. Cells were allowed to incubate for 48 hours in exosome-depleted cDMEM containing the HSA-mir-335 mimic or iEVs-335 (10^4/cell) as specified in the Material and Methods section in the paper. After 48 hours, cells were washed x3 and supplemented with fresh exosome-depleted cDMEM for 2, 4 or 8 days following wash and re-supplementation with fresh medium. Cells were collected and mRNA was extracted with RNAGEM tissue plus kit (Zygem, RTP0100). cDNA was generated from 100 µg of total RNA with High Capacity cDNA Reverse Transcription Kit (Thermo-Fisher, 4368814). qPCR analysis of samples utilized primers for SOX4 target (Thermo-Fisher, Hs00268388_s1SOX4) and β-actin as endogenous control (Thermo-Fisher, Hs99999903_m1ACTB). Test were run in triplicate. Results (columns) are expressed as mean ± SD of RQ values. Numbers in parenthesis refer to percentage (%) reduction relative to untreated control of same day harvest.

SUPPLEMENTARY DATA

Extracellular vesicles produced in B cells deliver tumor suppressor miR-335 to breast cancer cells disrupting oncogenic programming *in vitro* and *in vivo*

Almanza, Rodvold, Tsui, Jepsen, Carter and Zanetti

Supplementary Tables

Supplementary Table 1: SOX4 genes. Summary of differential expression of SOX4 target genes between iEVs-335 treated LM2 samples and pooled control conditions. Genes are annotated with log2 fold change in expression, p-value and Benjamini-Hochberg adjusted p-value.

Supplementary Table 2: has-miR-335 target genes. Summary of differential expression of hsa-miR-335 target genes between iEVs-335 treated LM2 samples and pooled control conditions. Genes are annotated with log2 fold change in expression, p-value and Benjamini-Hochberg adjusted p-value.

Supplementary Table 3. Expression fold change and significance of change for genes in LM2 cells treated with iEVs-335 relative to pooled control conditions. Genes are annotated with log2 fold change in expression, p-value and Benjamini-Hochberg adjusted p-value.

Supplementary Table 4. Pathways enriched for genes perturbed by iEVs-335 treatment. Gene Set Enrichment Analysis was performed to identify pathways enriched for transcriptional changes caused by iEVs-335 treatment. Pathways identified at an FDR q-value < 0.05 were considered enriched for differentially expressed genes.

Supplementary Table 5. Analysis of hallmark cancer genes in the miR-335 treated condition relative to control. Hallmark cancer genes are those identified in Gao, S. et al. (*JAMA Oncol* **2**, 37-45 (2016). Pathways identified at an FDR q-value < 0.05 were considered enriched for differentially expressed genes.
